# Supplementary material for: IL3 Has a Detrimental Effect on Hematopoietic Stem Cell Self-Renewal in Transplantation Settings
Source: Int J Mol Sci. 2022 Oct 22;23(21):12736. doi: 10.3390/ijms232112736 (PMC9655151; doi:10.3390/ijms232112736)
Supplement: Supplementary file 1 [file ijms-23-12736-s001.zip › ijms-1910455-supplementary.pdf]

Supplementary Material

A

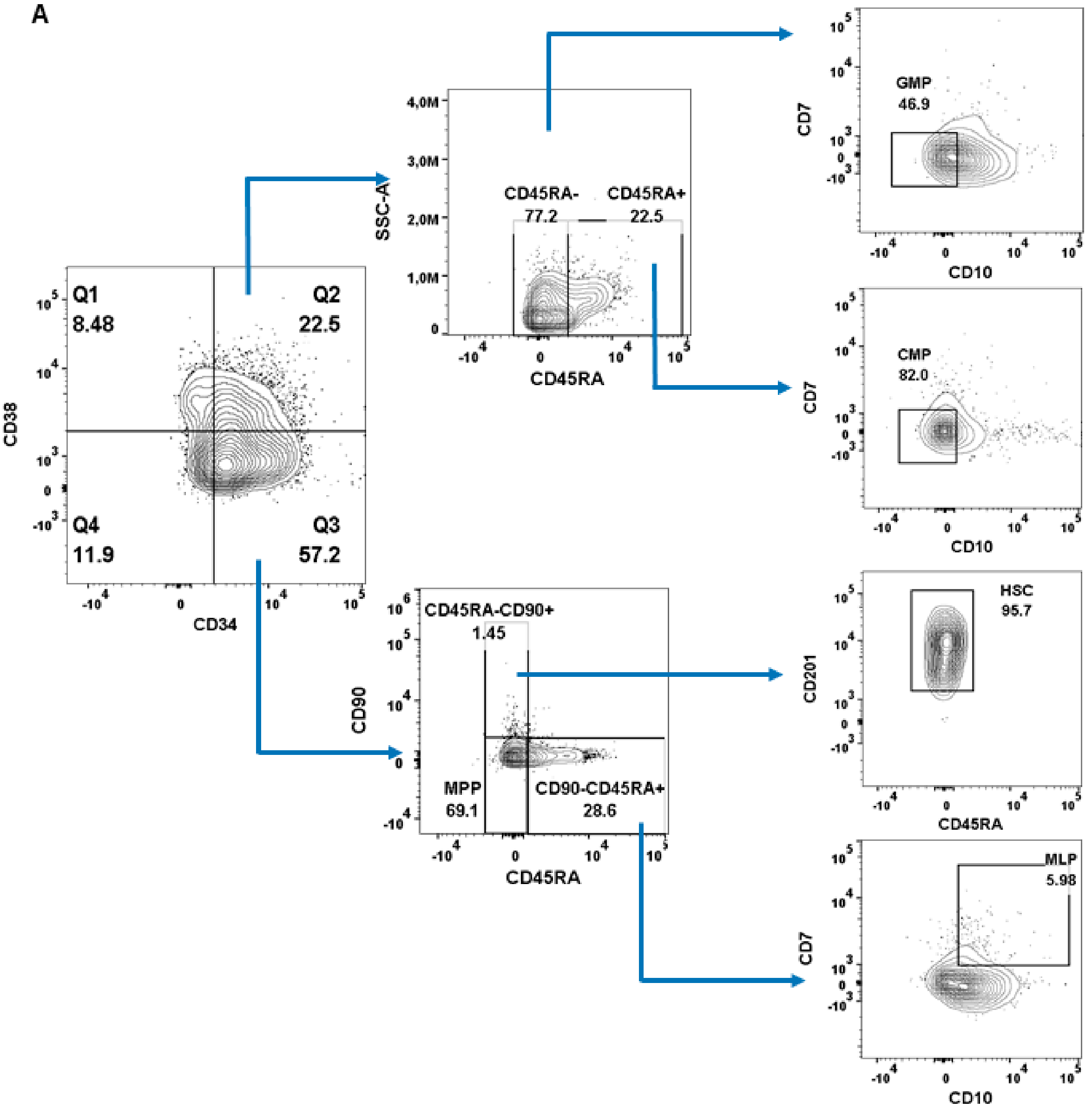

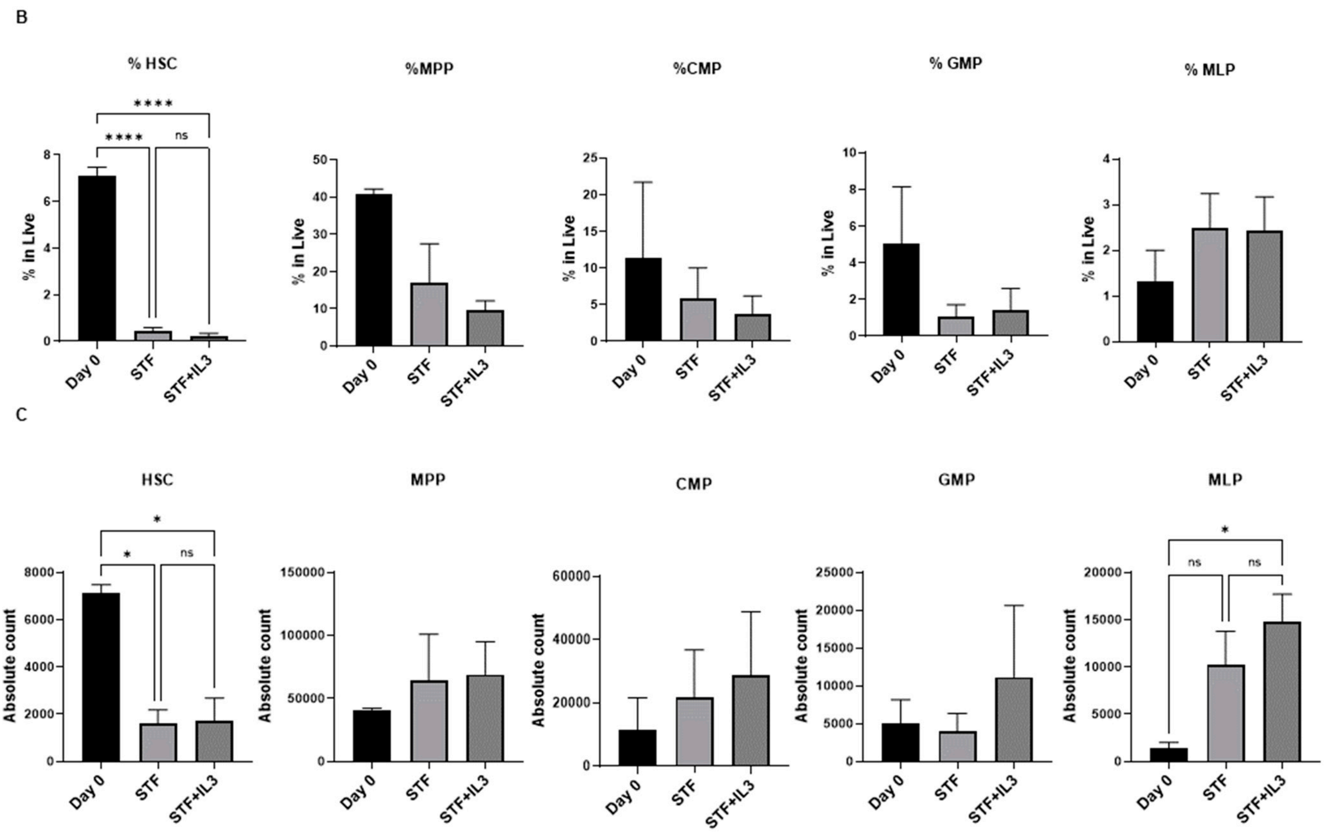

Supplementary Figure S1. Percentages and absolute count of hematopoietic stem cells and progenitors (HSPCs). (A) Representative gating strategy for phenotypic analysis of HSPCs. Hematopoietic stem cell (HSC): $CD34^{+}CD38^{-}CD90^{+}CD45RA^{-}CD201^{+}$, Multipotent Progenitor (MPP): $CD34^{+}CD38^{-}CD45RA^{-}CD90^{-}$, Multi-lymphoid Progenitor (MLP): $CD34^{+}CD38^{-}CD45RA^{+}CD90^{-}CD10^{+}CD7^{+}$, Common Myeloid Progenitor (CMP): $CD34^{+}CD38^{+}CD45RA^{+}CD10^{-}CD7^{-}$, Granulocyte-Macrophage Progenitor (GMP): $CD34^{+}CD38^{+}CD45RA^{-}CD10^{-}CD7^{-}$. (B) Percentage (C) and absolute cell counts of the different progenitor populations at day 0 and day 4 (n=3, *p<0,05, **p<0,01 and ***p<0,001, one-way Anova).

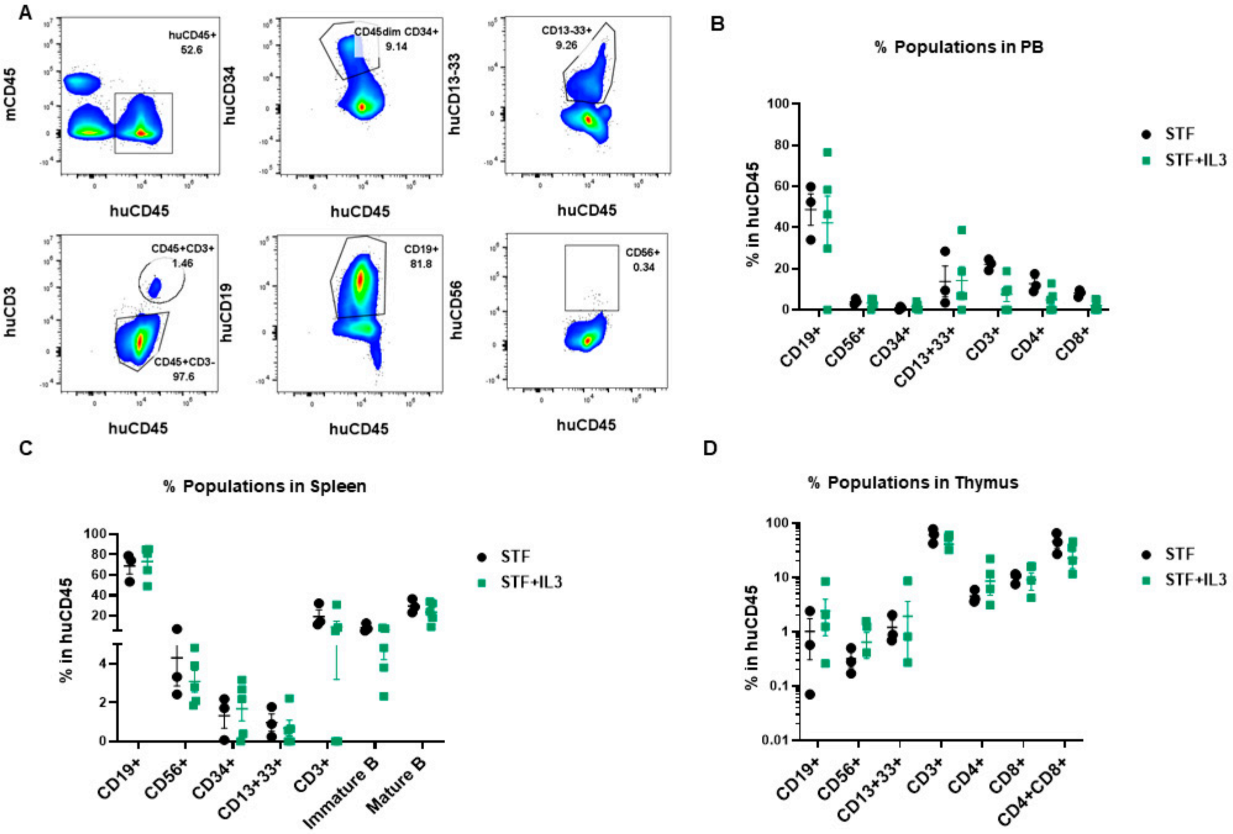

Supplementary Figure S2. Human engraftment in organs from primary transplanted NSG mice. (A) Representative of flow cytometry analysis strategy in bone marrow at week 20 post-transplantation of different populations within huCD45^{+}. (B-D) Percentages of different populations in PB, Thymus and Spleen.

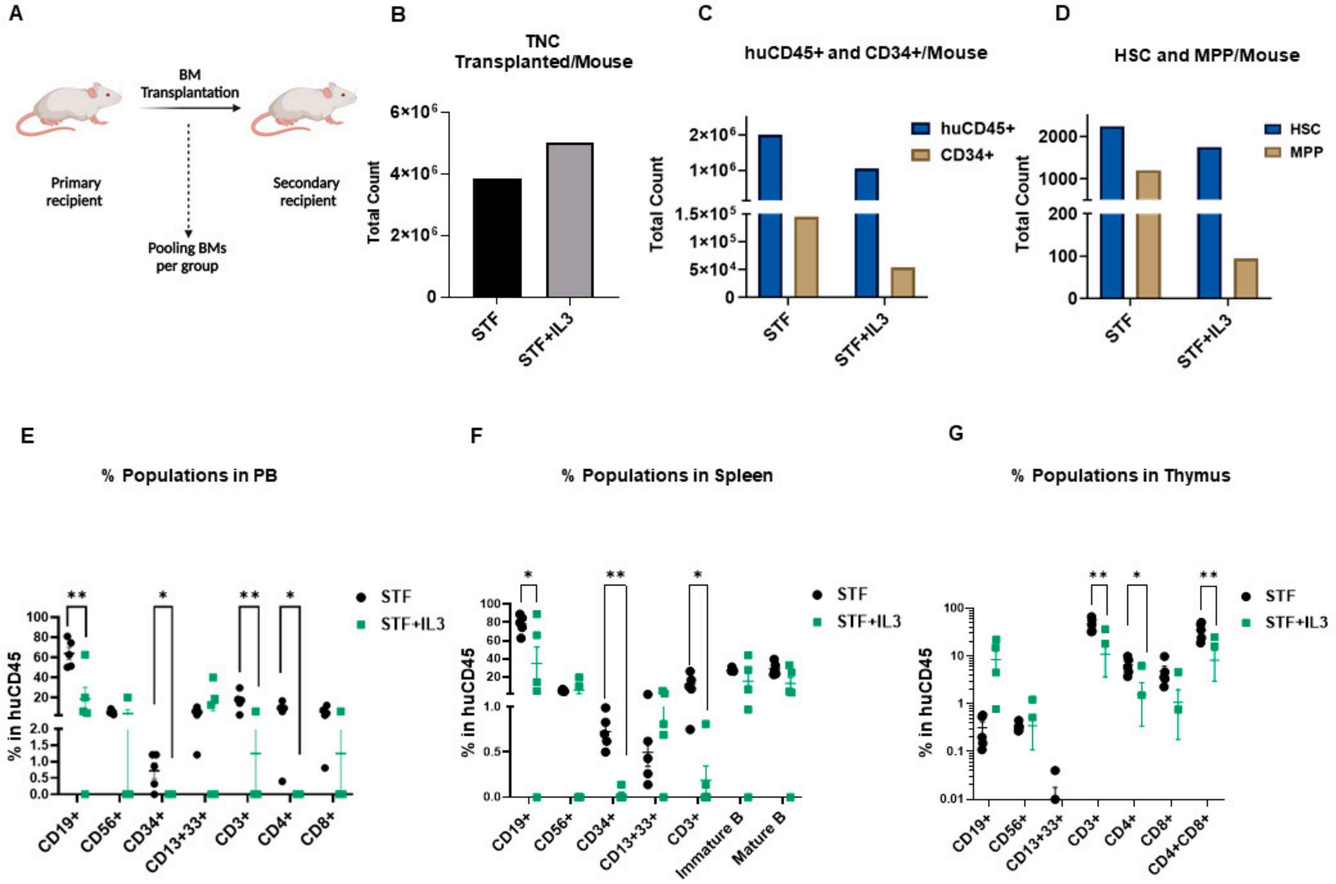

Supplementary Figure S3. Human engraftment in organs from secondary transplanted NSG mice. (A) Schematic picture of secondary transplantation. (B) Total nucleated cells (TNC) transplanted per mouse in each group. (C) Total count of huCD45^{+} and CD34^{+} cells that each mouse received per group. (D) Graph shows total count of HSC and MPP each mouse received per group at the time of secondary transplantation. (E-G) Engraftment of different populations in PB, Thymus and Spleen at week 20 post-transplantation (n=5; *p<0,05, **p<0,01 and ***p<0,001, unpaired t-test).

Name	Fluorochrome	Clone	Source
hCD1a	APC	HI149	BD Biosciences
hCD3	BV786	SK7	BD Biosciences
hCD4	BUV805	SK3	BD Biosciences
hCD5	BV480	UCHT2	BD Biosciences
hCD7	Pe-Cy5	CD7-6B7	Biolegend
hCD8	BV650	RPA-T8	Biolegend
hCD8	BUV496	RPA-T8	BD Biosciences
hCD10	APC-Cy7	HI10a	Biolegend
hCD13	Percp-Cy5.5	WM15	BD Biosciences
hCD19	PE-Cy7	HIB19	Invitrogen
hCD19	BV421	HIB19	Biolegend
hCD20	BV570	2H7	Biolegend
hCD33	Percp-Cy5.5	WM53	BD Biosciences
hCD34	Pe-CF594	581	BD Biosciences
hCD34	BV650	561	Biolegend
hCD38	Pe-Cy7	HIT2	Biolegend
hCD41	BV421	HIP8	Biolegend
hCD45	V450	HI30	BD Biosciences
hCD45RA	BV711	HI100	Biolegend
hCD45RA	BV510	HI100	BD Biosciences
hCD49f	PerCP-ef710	ebioGoH3	Invitrogen
hCD56	PE-Cy5	B159	BD Biosciences
hCD56	APC-Cy7	HCD-56	Biolegend
hCD62L	BV605	DREG-56	BD Biosciences
hCD71	AF700	M-A712	BD Biosciences
hCD90	APC	5 e10	Biolegend

hCD201	PE	RCR-401	Biolegend
hIgM	BV650	MHM-88	BD Biosciences
hIgD	BV480	IA6-2	BD Biosciences
hTCRgd	BV510	11F2	BD Biosciences
hTCRab	PE	MHM-88	BD Biosciences
mCD45	FITC	30-F11	BD Biosciences
Zombie NIR	NIR	-	Biolegend

Supplementary Table S1: List of antibodies used.
